# Mood as Representation of Momentum

**DOI:** 10.1016/j.tics.2015.07.010

**Published:** 2016-01

**Authors:** Eran Eldar, Robb B. Rutledge, Raymond J. Dolan, Yael Niv

**Affiliations:** 1 Wellcome Trust Centre for Neuroimaging, University College London, London WC1N 3BG, UK; 2 Max Planck University College London Centre for Computational Psychiatry and Ageing Research, London WC1B 5EH, UK; 3 Princeton Neuroscience Institute and Psychology Department, Princeton University, Princeton, NJ 08544, USA

**Keywords:** Mood, reinforcement learning, decision making

## Abstract

Experiences affect mood, which in turn affects subsequent experiences. Recent studies suggest two specific principles. First, mood depends on how recent reward outcomes differ from expectations. Second, mood biases the way we perceive outcomes (e.g., rewards), and this bias affects learning about those outcomes. We propose that this two-way interaction serves to mitigate inefficiencies in the application of reinforcement learning to real-world problems. Specifically, we propose that mood represents the overall momentum of recent outcomes, and its biasing influence on the perception of outcomes ‘corrects’ learning to account for environmental dependencies. We describe potential dysfunctions of this adaptive mechanism that might contribute to the symptoms of mood disorders.

## Trends

With increasing use of computational models to understand human behavior, scientists have begun to model the dynamics of subjective states such as mood.

Recent data suggest that mood reflects the cumulative impact of differences between reward outcomes and expectations.

Behavioral and neural findings suggest that mood biases the perception of reward outcomes such that outcomes are perceived as better when one is in a good mood relative to when one is in a bad mood.

These two lines of research establish a bidirectional interaction between mood and reinforcement learning, which may play an important adaptive role in healthy behavior, and whose dysfunction might contribute to psychiatric disorders.

## Why Do We Have Moods?

The enormous and disruptive impact of mood disorders in society 1, 2 might suggest that **mood** (see Glossary) is an evolutionary relic that may have been advantageous for early humans but impedes adaptive behavior in the modern world. Indeed, we often attribute irrational behavior to the emotional state of a person 3, 4, 5, 6. Our language also reflects this view, with expressions such as ‘moody’ and ‘being in a mood’ carrying negative connotations. We argue that moods serve an important role in adaptive behavior, even in the modern world. We elucidate this role by considering recent findings regarding the dynamics of mood, as well as its interaction with the processes of learning and decision making. Based on these findings, we propose that moods benefit ‘moody’ agents by mitigating inefficiencies that can arise in the process of learning about the natural environment.

Advances in computational modeling have greatly facilitated an understanding of how humans learn from **outcomes** to make better decisions 7, 8, 9. Recently, scientists have begun to utilize the same computational framework to study the dynamics of human emotional states in health and in mental disorders, focusing on how these states affect and are affected by learning and decision-making processes 10, 11, 12. In particular, two burgeoning lines of research have sought to characterize precisely, on the one hand, the causes of moods, and on the other the consequences of mood states for learning and decision making. We first review these two largely separate strands of research and then integrate them within a coherent theoretical framework. We propose that mood represents the overall momentum of reward in the environment, and that this representation serves to facilitate efficient learning by accounting for statistical dependencies in the availability of rewards that are prevalent in nature.

## Causes: Mood Depends on the Cumulative Impact of Unexpected Outcomes

To understand the function of mood, we first need to consider its causes. A vast psychological literature demonstrates that mood can be manipulated via a range of techniques [13]. Presentation of a film or story with emotional content is a common and effective mood-induction technique. Other stimuli that reliably affect mood include music, self-referential statements, observed social interactions, and facial expressions. While these stimuli are easy to present in laboratory experiments, they are not readily quantifiable and are typically applied categorically, without variation in either quantity or intensity. Monetary outcomes, by contrast, can be precisely controlled and have also been shown to affect mood 14, 15.

Another line of research, originating primarily in an economics literature, considers real-world circumstances that covary with subjective well-being [16]. Such research is inherently correlational, but has identified various factors that impact on mood, including outcomes of sporting events and levels of sunshine 17, 18. Moreover, to measure the dynamics of emotional state that are relevant to understanding adaptive behavior, well-being researchers have developed experience-sampling techniques that probe participants as to their current subjective state while they go about their daily lives 19, 20. These techniques, which involve periodically asking participants about their current emotional state and what they are doing, are considered the ‘gold standard’ for investigating real-world emotion. Experience-sampling and related methods, such as the day-reconstruction method 21, 22, show that in typical individuals some activities (e.g., conversation, eating) are consistently related to higher happiness ratings, while other activities (e.g., work, commuting) are consistently related to lower happiness ratings. Some studies have also applied these methods to study differences in well-being across individuals, showing greater mood instability in bipolar disorder 23, 24 and greater negative affect in depression [25] compared to healthy subjects.

Recent research has used experience sampling to examine momentary mood fluctuations during a laboratory-based probabilistic reward task in which monetary rewards varied from trial to trial [26]. The main conclusion of the study was that happiness depends not on how well things are going (in terms of cumulative earnings) but whether they are going better than expected. In particular, self-reported happiness depended on ‘reward prediction errors’ (RPEs; Box 1), that is, the difference between expected outcomes and obtained outcomes. The laboratory results were also replicated in a large-scale smartphone-based experiment with 18420 participants. In addition, blood-oxygen-level dependent (BOLD) activity measured using functional magnetic resonance imaging (fMRI) in the ventral striatum, a target area for dopamine neurons that represent RPEs 27, 28, 29, 30, 31, 32, 33, correlated with RPEs and with subsequent happiness ratings. This is consistent with a possible role for dopaminergic RPE signals in determining mood. Indeed, pharmacologically boosting dopamine levels has recently been shown to increase the happiness that results from particular types of reward [34].

## Consequences: Mood Biases Perception of Outcomes

**Figure 1:**
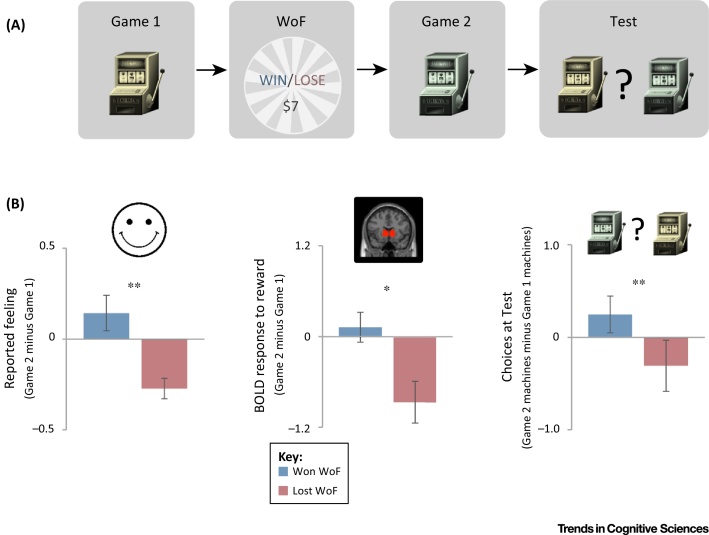
The Effect of a Monetary Outcome on Mood and on Subsequent Neural and Behavioral Responses to Rewards. (A) Experimental design of [38]. To manipulate mood, a one-shot wheel-of-fortune (WoF) draw was held in between games, resulting in a gain or loss of $7. Game 1 and Game 2 involved different sets of slot machines with similar reward probabilities, and participants learned about the machines by trial and error. In the Test phase, participants chose between slot machines from Game 1 and Game 2. (B) In participants who reported high emotional instability, the WoF outcome affected self-reported mood (left, *n* = 28 per group; 1 is maximally happy and −1 is maximally unhappy) and striatal BOLD response to reward measured by fMRI (middle, *n* = 13 per group) during Game 2 as compared to Game 1 (shown are *t* values). In the test phase, those participants who experienced a WoF win preferred Game 2 machines, which they had played while in a better mood. By contrast, participants who had experienced a WoF loss preferred Game 1 machines, which they played before the WoF draw (right, *n* = 28 per group; 1 indicates complete preference for Game 2 machines). * *P* < 0.05, ** *P* < 0.001. Adapted from [38].

It has long been thought that happiness induces a ‘rosy’ perspective, whereas a depressed mood engenders negative judgments 35, 36, 37. More recently, researchers have used computational methods in laboratory experiments to precisely quantify the effects of emotional state on behavior. In one study [38], mood was manipulated using a wheel-of-fortune draw in which participants either won or lost a relatively large sum of money. In participants independently identified as being less emotionally stable, winning the draw increased self-reported happiness and the effect of subsequent rewards on subsequent choices. By contrast, losing the draw reduced happiness, as well as neural responses to subsequent rewards, and the effect of those rewards on choices (Figure 1). Manipulating mood by viewing emotional facial expressions is also known to induce a bias in both neural responses to rewards [39] and learning from rewards [40]. Moreover, a depressed mood is associated with a reduced effect of rewards on subsequent choices 41, 42, an effect that is better explained by reduced valuation of reward than by a reduced rate of learning [43]. A similar relationship may also hold between an anxious emotional state and perception of aversive outcomes: stressed humans and rats respond, neurally and behaviorally, to aversive outcomes and ambiguous stimuli as if they are worse than they actually are 44, 45, 46.

Other studies have explored additional effects of mood on decision making, many of which can be similarly understood as reflecting a biased perception of reward or of stimuli indicating reward availability. For example, positive mood induces risk-taking in laboratory experiments 47, 48 and in real financial markets 49, 50, possibly by biasing upwards the perceived probability of future positive outcomes [51]. In addition, repeated positive RPEs, which should improve mood [26], invigorate reward-seeking behavior 52, 53, 54, 55, possibly reflecting an implicit belief in greater reward availability. Furthermore, a positive emotional state reinforces, and a negative emotional state inhibits, one's current mode of thought, presumably by biasing perception of how well that mode of thought is functioning 56, 57, 58. Finally, many studies suggest that a depressed mood is associated with greater attention or sensitivity to negative information, an effect that may underlie biased perception of outcomes. Notably, both effects can be seen to reflect an implicit belief that things are worse than the objective evidence suggests 59, 60.

The upshot of this research is that mood induced by a stimulus can affect judgment about other, potentially unrelated, stimuli. Indeed, this property may have given mood its reputation as a rich fountain for irrational behavior. Any attempt to rationalize moods must therefore explain how such biased judgments, which in some cases may reinforce irrelevant actions, nevertheless promote adaptive behavior.

## The Function of Mood

According to current theories, agents can maximize reward by keeping track of how much reward is obtained in each experienced state of the environment, and then choosing actions that return them to the states in which such reward has been most abundant 7, 8. For example, an animal using such a mechanism can learn which specific trees bear more fruit and focus its foraging efforts accordingly. This type of ‘**reinforcement learning**’ algorithm [9] constitutes a powerful way to learn about the environment and converges upon optimal behavioral policies (e.g., [61]). However, there are many real-world situations for which such an algorithm may be poorly equipped. We propose that the information represented by mood is used to mitigate problems that arise in the application of reinforcement learning to such real-world problems.

One such learning inefficiency arises when changes in reward in different states are correlated. For instance, increased rainfall or sunshine may cause fruit to become more abundant in all trees simultaneously. In this situation, it makes little sense to update expectations for each tree independently, and a more efficient learning algorithm would instead infer a general increase in reward and update expectations for all related trees accordingly. We suggest this is the function of mood. If fruit becomes more abundant in all trees, a foraging animal will be positively surprised multiple times as it visits adjacent trees and, as a result, its mood will improve. Improved mood will bias the subjective reward for each subsequent fruit upwards, and because these observations are used to update expectations, expectations associated with these trees will be adjusted upwards more rapidly than they would be otherwise. In essence, the effect of positive surprises will be enhanced as more positive surprises are encountered.

Through the existence of mood, as an animal learns from experience, its expectations come to reflect not only the reward associated with each particular state (e.g., each tree), but also recent overall changes in the availability of reward in its environment. In this way, learning can account, albeit approximately, for the impact of multiple general environmental factors without having to directly infer the number of factors or the extent of their impact (Box 2). We have described one scenario in which this can be beneficial, but such a generalization mechanism can improve the efficiency of learning in any environment in which different sources of reward are interdependent. Indeed, such interdependencies may be the rule rather than the exception, for both animals and humans, because success in acquiring skills, material resources, social status, and even mating partners can be tightly correlated.

Mood can also be useful for learning in another common scenario in which current changes in reward predict later changes in reward. Many processes in the natural world have such momentum. For instance, initial increases in fruit availability may indicate that spring is coming and that further increases are probable. In such a case, a positive mood would represent inference of a positive momentum – which would, in turn, bias perception of subsequent rewards upwards. Because rewards would then be perceived as better than they really are, expectations would be updated upwards quickly and would catch up with rising rewards. Similarly, if reward availability is decreasing in an environment (e.g., winter is coming), then a negative mood leads to rewards being perceived as less good than they actually are (even though increasingly rare rewards still result in positive RPEs) and expectations will catch up with declining rewards, allowing behavior to be quickly adjusted (e.g., hibernate). In accordance with this idea, the relationship between mood and reward perception suggested by the recent literature can be formally derived as statistical inference of average reward and its momentum (Box 2).

## From Function to Dysfunction

**Figure 2:**
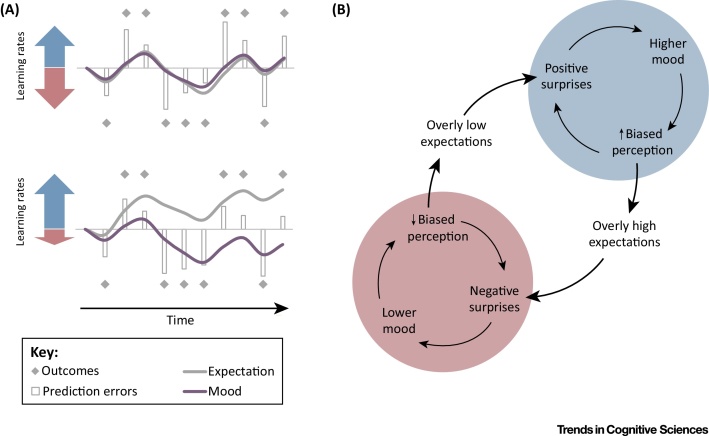
Schematic of Possible Mood Dysfunctions. (A) (Top) Given a similar rate of learning in response to positive and negative outcomes, an environment in which positive and negative outcomes are equally likely leads to neutral expectations and a neutral mood on average. (Bottom) A lower rate of learning from negative outcomes leads to optimistic expectations and therefore larger negative prediction errors and persistent negative mood, a symptom seen in major depressive disorder. (B) Escalatory positive-feedback dynamics might turn mood into a ‘self-fulfilling prophecy’, leading to emotional instability, a major symptom of bipolar disorder. Positive surprises improve mood, biasing perception of outcomes upwards, thereby increasing the frequency and magnitude of further positive surprises. Optimistic expectations develop owing to the biased perception of outcomes. Mood stabilizes once expectations catch up with perceived outcomes, but subsequent outcomes, whose perception in now unbiased, then tend to fall short of optimistic expectations. Thus, negative surprises follow, thereby diminishing mood and biasing perception of outcomes downward. Similar positive-feedback dynamics then engender pessimistic expectations, setting the stage for the cycle to repeat, oscillating between good and bad mood indefinitely even if there are no changes in the actual distribution of outcomes.

Identifying the function of mood points to how it might be compromised, potentially leading to maladaptive behavior. The proper function of mood, as we delineate, increases the efficiency of learning about the environment when emotional reactions to changes in reward are appropriate in intensity and duration. Positive or negative moods maximize their usefulness by persisting only until expectations are fully updated in accordance with changes in rewards. Indeed, happiness eventually returns to a baseline level even following highly significant changes in circumstances [62], including winning the lottery [63], whereas excessive happiness can induce maladaptive behavior 64, 65. This homeostasis crucially depends on appropriate updating of expectations, that is, on the integrity of learning processes. If, for instance, expectations are not updated downwards following outcomes that are worse than expected, encountering the same outcomes again would continue to generate negative surprises indefinitely, inducing a negative mood. In fact, in environments with even modest amounts of variability or randomness, it suffices that the rate of learning (*η*_*t*_ in Box 2) is lower for negative than for positive surprises in order for overly optimistic expectations to develop. As a result, the frequency and magnitude of negative surprises would increase, leading to low mood (Figure 2A). Indeed, low serotonin function, which has been associated with impaired learning from negative outcomes [66], is linked to both depression and risk-taking behavior [67], two co-occurring conditions 68, 69, 70, 71 that may stem from lower negative learning rates and consequent overly optimistic expectations [30]. Interestingly, in the general population, positive mood and risk aversion predominate 72, 73, possibly indicating higher learning rates for negative than for positive surprises, which could reflect the greater importance to survival of avoiding negative outcomes.

More generally, if a negative mood is too intense or persists for too long, positive feedback dynamics can exacerbate the situation. Bad mood will result in subsequent outcomes being perceived as worse than they really are, leading to further negative surprises that induce further decreases in mood, which in turn will make outcomes seem even worse, and so on (Figure 2B). As expectations are updated to match biased perception of outcomes, overly pessimistic expectations can develop. Only if expectations catch up with perceived outcomes will the escalatory dynamics abate and de-escalation begin. Empirical findings indicate that an affective perceptual bias precedes ostensible changes in mood in response to treatment with serotonergic drug in major depressive disorder [74], an observation that supports a possible role for such a feedback cycle in the unfolding of depressive episodes.

If mood does eventually return to baseline levels, the pessimistic expectations that developed when mood was lower may now lead to increased positive surprises and improved mood. In some individuals, good mood may also persist and a positive feedback cycle may develop in the opposite direction, with good mood biasing perception of outcomes upwards, thereby increasing positive surprises, which further improve mood (Figure 2B). Overly optimistic expectations will develop, setting the stage again for negative surprises, which decrease mood, and potentially turning the cycle in the negative direction again. The overall result could be oscillatory dynamics, as observed in bipolar disorder, in which expectations and mood cyclically fluctuate even in the absence of objective changes in the external environment.

Thus, while learning makes outcomes more predictable and promotes habituation to these outcomes, the biasing effect of mood on the perception of outcomes has the opposite sensitizing effect of increasing responsivity to outcomes. A predisposition to emotional instability could therefore result from any factor that strengthens the sensitizing effect of mood or that weakens the habituating effects of learning (e.g., *η*_*t*_ << $\eta_{t}^{'}$ and high *f*_*t*_ in Box 2). Laboratory evidence suggests that such sensitization may indeed underlie emotional instability. Specifically, participants who report being more emotionally unstable show stronger effects of outcomes on their feelings, as well as on their evaluation of subsequent outcomes [38]. It is notable that clinically pathological escalation in the direction of negative mood seems to be more prevalent than escalation of positive moods. Negative moods might escalate more frequently because of a stronger biasing effect, possibly reflecting the greater overall adaptive significance of reacting quickly to negative changes in momentum.

## Concluding Remarks

We have outlined a normative perspective on mood, according to which mood serves as a representation of the momentum of changes in reward. This momentum signal can be used to adjust learning to account for dependencies between different states and across time. How this momentum is represented in the brain is an open question (see Outstanding Questions), although some studies implicate the neuromodulators serotonin and dopamine 26, 27, 53, 75, 76. Our approach suggests different ways in which the function of mood might be disrupted, and we have described two specific dysfunctions that might contribute to the emergence of depression and mood instability. The proper function of mood might also lead to maladaptive behavior in particular scenarios. Thus, moods can reflect inference of momentum even when there is none in the environment, leading to excessive optimism or pessimism. However, the ubiquity of moods and the extent of their impact on our lives tells us that, throughout the course of evolution, our moodiness must have conferred a significant competitive advantage. Being moody at times may be a small price to pay for the ability to adapt quickly when facing momentous environmental changes.

Outstanding Questions How is mood represented in the brain?

How do long-lasting moods interact with and relate to more short-lasting emotions?

Can an anxious mood be understood as a representation of momentum in aversive outcomes?

How can our model, which was derived from studies of healthy individuals, be utilized to explain the dynamics of mood in psychiatric mood disorders?

How do antidepressants, mood stabilizers, and other therapeutic interventions affect the dynamics of mood?

## Figures and Tables

**Figure I:**
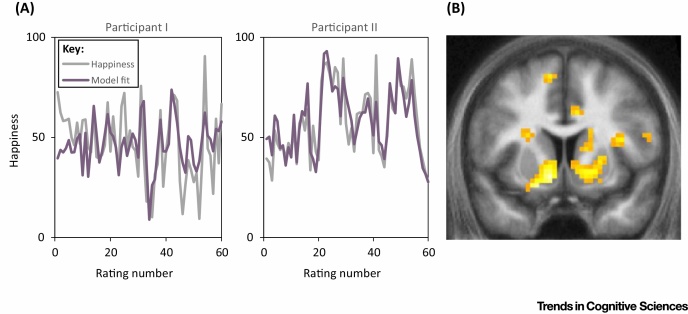
Subjective Well-Being, Model Predictions, and Neural Activity. (A) Happiness ratings during a probabilistic reward task for two example participants. The model predicts ratings based on the past history of expectations and reward prediction errors (RPEs) resulting from those expectations. (B) BOLD activity in the ventral striatum measured by fMRI was correlated with subsequent happiness ratings, consistent with striatal representation of RPEs contributing to changes in mood. Adapted from [26].

**Figure I:**
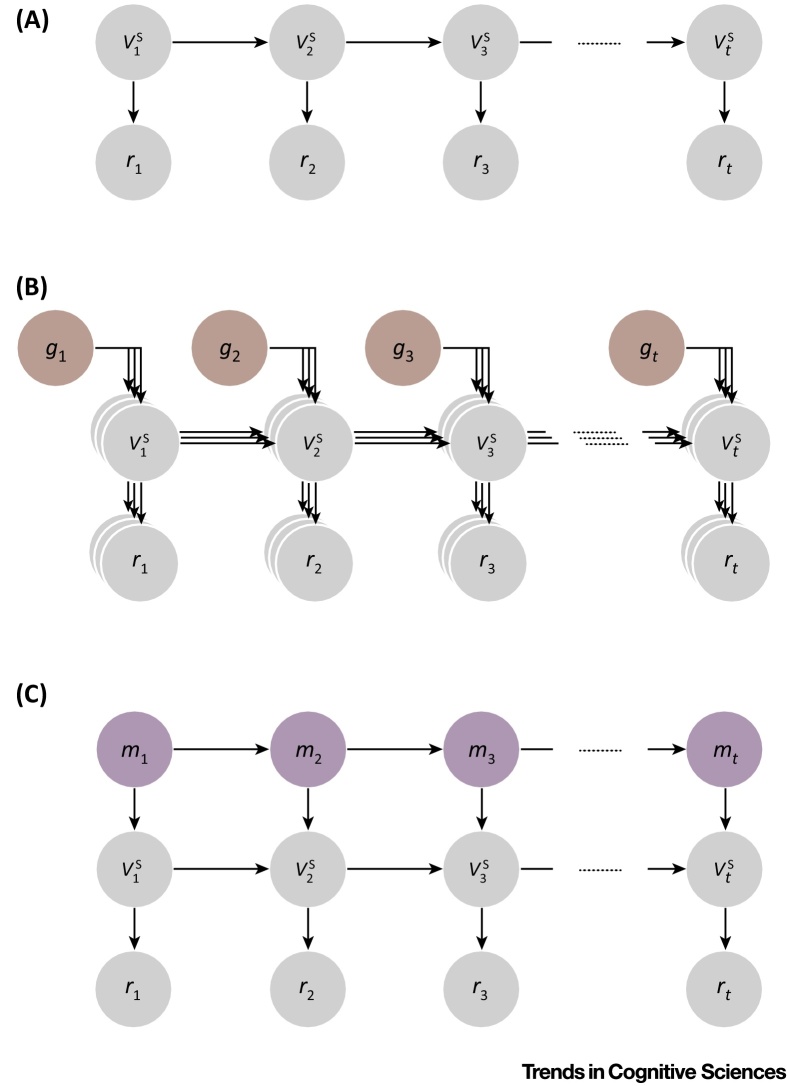
Probabilistic Kalman–Filter [80] Models of the Environment. The reward outcome *r* at time-step *t* is sampled from a normal distribution whose mean $v_{t}^{s}$ is specific to the current state. (A) For a particular state, depicted here, changes in the mean follow a random walk with normally distributed noise. (B) A general environmental factor affects multiple states. At each time-step *t*, a general factor *g_t_* is sampled from a normal distribution whose mean is zero, and is then added to multiple state means ($v_{t}^{s}$). (C) Changes in reward follow an underlying momentum. The mean reward $v_{t}^{s}$ of a state is sampled from a normal distribution whose mean is the sum of the previous mean $v_{t-1}^{s}$ and the current momentum *m_t_*. Changes in momentum follow a random walk.

## Box 1 A Computational Model of Momentary Subjective Well-Being

Mood is thought to reflect both positive and negative outcomes that have been recently experienced. However, a recent study demonstrated that reported happiness during value-based decision making specifically depends on reward expectations and how actual outcomes differ from these expectations [26]. Subjects repeatedly chose between (i) outcomes that were certain and (ii) gambles with systematically varying potential gains and losses. In addition, they were asked ‘how happy are you at this moment?’ after every 2–3 trials. Happiness ratings were modeled as follows (Figure I):

$${\text{Happiness}}_{t}=w_{0}+w_{1}\sum_{j=1}^{t}\gamma^{t-j}{\text{CR}}_{j}+w_{2}\sum_{j=1}^{t}\gamma^{t-j}{\text{EV}}_{j}+w_{3}\sum_{j=1}^{t}\gamma^{t-j}{\text{RPE}}_{j}.$$

For each trial *j* (from the first trial and up to the current trial *t*), if the certain reward was chosen it was entered into the equation as CR_*j*_. Conversely, if the gamble was chosen two terms were entered into the equation: EV_*j*_, the expected value of the gamble, and RPE_*j*_, the difference between the actual outcome and the gamble EV. The weights *w* (which include a constant term *w*_0_) capture the influence of task variables on momentary happiness. These influences decay exponentially in time with a forgetting factor 0 ≤ *γ* ≤ 1 such that recent events are more influential than earlier events. Model parameters were significantly positive on average in three laboratory experiments and in a large-scale smartphone-based field study. RPE weights were significantly higher than EV weights, showing that surprise about outcomes had a stronger effect on happiness than expectations about outcomes. However, changes in the two other task variables (CR and EV) also reflect surprise about the certain rewards and gambles that were made available, and can also be thought of as a type of RPE. Therefore, these results suggest that happiness reflects a running average of recent RPEs in which different types of prediction errors may be differently weighted.

## Box 2 Different Learning Algorithms for Different Environments

The optimal learning algorithm for a particular environment can be determined by creating a probabilistic model of the environment and then using the laws of probability (specifically, Bayes rule) to infer what outcomes are most likely given previously observed events 77, 78. For example, if reward in the environment is determined by the state we are in, and states are independent of one another (Figure IA), the optimal learning algorithm estimates the reward expected in each state similarly to a standard reinforcement learning algorithm [79]:

$$v_{t+1}^{s}=v_{t}^{s}+\eta_{t}(r_{t}-v_{t}^{s})$$

That is, the estimated mean reward *v*^*s*^ at state *s* is updated at each time-step *t* according to the difference between the observed reward *r* and the previous estimate (i.e., the prediction error) scaled by a learning rate *η*_*t*_.

If, however, different states are not independent, but instead multiple states are similarly affected by general environmental factors (Figure IB), then an efficient learning algorithm would update its expectations of all states that are affected by the same factor when experiencing a prediction error in any one of them. This might not be feasible with an unknown number of general factors, each applying to only a subset of neighboring states (e.g., the abundance of fruit is more tightly correlated for trees growing in the same valley). However, a simple approximation is to keep track of all prediction errors in recently visited states:

$$m_{t+1}=m_{t}+{\eta^{\prime }}_{t}((r_{t}-v_{t}^{s})-m_{t})\text{.}$$

where ${\eta^{\prime }}_{t}$ is a learning rate, and to assume that other states that are close in space or time have also changed similarly. One way to implement this solution is to bias the perception of outcomes in subsequent states by adding a bonus to the actual reward that reflects the tracked average of recent prediction errors (*m*_*t*_) such that this bonus gets incorporated into learned expectations:

$$v_{t+1}^{s}=v_{t}^{s}+\eta_{t}(f_{t}\cdot m_{t}+r_{t}-v_{t}^{s})\text{.}$$

where *f*_*t*_ is a scaling factor. This way expectations of reward in particular states come to reflect not only the outcomes experienced in those states, but also outcomes experienced in other related states.

The above algorithm can also be useful with only a single state, when changes in rewards are not independent in time but instead follow an underlying momentum (Figure IC). In this case, precise inference requires estimation of the underlying momentum, which again takes the form of a running average of recent prediction errors. This average can then be integrated in the expectation update equation as above to account for the dependency between adjacent time-steps (see Note S1 in the supplemental information online for mathematical derivations).

## Glossary

Mood: ‘moods’ differ from ‘emotions’ in that moods typically last longer. In addition, while an emotion typically relates to a single stimulus, moods are less tightly linked to particular events and can reflect the cumulative impact of multiple stimuli. Moods influence a threshold for elicitation of emotion, for example, depressed mood can facilitate the expression of an emotion of anger. Thus, many researchers consider emotions and moods as parallel interacting processes that take place over different timescales. Emotional states can be measured along different dimensions. We focus on the valence dimension of happiness versus unhappiness.
Outcome: an outcome is any event of motivational significance. Outcomes can be appetitive or aversive. In this article we focus on reward outcomes that are monetary gains and losses because these outcomes can be precisely manipulated and quantitatively related to both mood and behavior.
Reinforcement learning: a class of algorithms that learn from trial and error to predict which states of the environment and which actions in those states will maximize cumulative future reward and minimize cumulative future punishment.
