# Supplementary material for: Mood as Representation of Momentum
Source: Trends Cogn Sci. 2016 Jan;20(1):15–24. doi: 10.1016/j.tics.2015.07.010 (PMC4703769; doi:10.1016/j.tics.2015.07.010)
Supplement: Supplementary file 1 [file mmc1.pdf]

Supplementary Material

Mood as representation of momentum

Eran Eldar[1,2,*,†], Robb B. Rutledge[1,2,†], Raymond J. Dolan[1,2], Yael Niv[3]

[1] Wellcome Trust Centre for Neuroimaging, University College London, London WC1N 3BG, UK
[2] Max Planck University College London Centre for Computational Psychiatry and Ageing Research, London WC1B 5EH, UK
[3] Princeton Neuroscience Institute and Psychology Department, Princeton University, Princeton, NJ 08544, USA
[*] Correspondence: e.eldar@ucl.ac.uk (E. Eldar).
[†] These authors contributed equally to this article.

Supplementary Material Inventory:

1. Supplementary Note S1

Supplementary Note S1. Derivation of optimal learning algorithms.

Here we will derive the optimal learning algorithm for the models in Box 2 by casting each model as a Kalman filter model [S1], and then using the standard solution for Kalman filters.

A Kalman filter model consists of an unobservable (hidden) state $\mathbf{x}$ and state covariance matrix $\mathbf{P}$, which need to be inferred from a sequence of observations $\mathbf{r}$, and a set of assumptions about how observations are generated. These assumptions include the state-transition matrix $\mathbf{F}$, which determines how states change over time; the state-to-observation transformation matrix $\mathbf{H}$, which determines how observations are generated given the current state; and the state noise covariance matrix $\mathbf{Q}$ and observation noise covariance matrix $\mathbf{C}$, which introduce stochasticity into state transitions and the generation of observations.

Given observation $\mathbf{r}_t$ at time step t, an optimal learning algorithm updates the estimates of the current state and the state covariance matrix as follows:

$$\mathbf{x}_t = \mathbf{F}\mathbf{x}_{t-1} + \mathbf{K}_t(\mathbf{r}_t - \mathbf{H}\mathbf{F}\mathbf{x}_{t-1}) \quad (1)$$

$$\mathbf{P}_t = \mathbf{F}\mathbf{P}_{t-1}\mathbf{F}^{\mathrm{T}} + \mathbf{Q} - \mathbf{K}_t\mathbf{H}(\mathbf{F}\mathbf{P}_{t-1}\mathbf{F}^{\mathrm{T}} + \mathbf{Q}), \quad (2)$$

where

$$\mathbf{K}_t = (\mathbf{F}\mathbf{P}_{t-1}\mathbf{F}^{\mathrm{T}} + \mathbf{Q})\mathbf{H}^{\mathrm{T}}(\mathbf{H}(\mathbf{F}\mathbf{P}_{t-1}\mathbf{F}^{\mathrm{T}} + \mathbf{Q})\mathbf{H}^{T} + \mathbf{C})^{-1} \quad (3)$$

<u>Model I: Independent states</u>

In this baseline model, described in Box 2 (Figure IA), each state corresponds to an independent one-dimensional Kalman filter model with the following properties:

$$\mathbf{x}_t = [v_t] \qquad \mathbf{F} = [1] \qquad \mathbf{H} = [1] \qquad \mathbf{Q} = [q]$$

Substituting the above terms into equations (1) and (3) we get:

$$v_t = v_{t-1} + K_t(r_t - v_{t-1}) \quad (4)$$

where

$$K_t = \frac{P_{t-1} + q}{P_{t-1} + q + C} \quad (5)$$

Thus, the estimated state is updated by the difference between the observation and the previous estimate (i.e., the prediction error; $r_t - v_{t-1}$) multiplied by an adaptive learning rate (K_t) that reflects the relationship between the state and observation variance.

Model II: States with independent and shared variance

The next model described in Box 2 (Figure IB) consists of multiple states, all of which are affected by the same general source of variance. For the case of three states, the model corresponds to a three-dimensional Kalman filter whose three dimensions represent three states with both state-specific ($\mathbf{q_{1:3}}$) and shared (q_g) variance:

$$\mathbf{x}_t = \begin{bmatrix} v_{t,1} \\ v_{t,2} \\ v_{t,3} \end{bmatrix} \qquad \mathbf{F} = \begin{bmatrix} 1 & 0 & 0 \\ 0 & 1 & 0 \\ 0 & 0 & 1 \end{bmatrix} \qquad \mathbf{H} = \begin{bmatrix} 1 & 0 & 0 \\ 0 & 1 & 0 \\ 0 & 0 & 1 \end{bmatrix} \qquad \mathbf{Q} = \begin{bmatrix} q_g + q_1 & q_g & q_g \\ q_g & q_g + q_2 & q_g \\ q_g & q_g & q_g + q_3 \end{bmatrix}$$

Substituting the above terms into equations (1) and (3) we get:

$$\begin{bmatrix} v_{t,1} \\ v_{t,2} \\ v_{t,3} \end{bmatrix} = \begin{bmatrix} v_{t-1,1} \\ v_{t-1,2} \\ v_{t-1,3} \end{bmatrix} + \mathbf{K}_t \begin{bmatrix} r_{t,1} - v_{t-1,1} \\ r_{t,2} - v_{t-1,2} \\ r_{t,3} - v_{t-1,3} \end{bmatrix}, \quad (6)$$

where

$$\mathbf{K}_t = (\mathbf{P}_{t-1} + \mathbf{Q})(\mathbf{P}_{t-1} + \mathbf{Q} + \mathbf{C})^{-1} \quad (7)$$

Thus, the estimated states are updated by the vector of prediction errors ($\mathbf{r}_t - \mathbf{x}_{t-1}$) multiplied by the matrix $\mathbf{K}_t$, which integrates the different types of covariance. Importantly, due to the variance that is shared between the states (i.e., the off-diagonal entries in $\mathbf{Q}$), the Kalman gain matrix $\mathbf{K}_t$ has nonzero off-diagonal values, and thus each state is updated not only by its own prediction error, but also by the prediction errors of the other states.

Model III: A state with momentum

The final model described in Box 2 (Figure IC) corresponds to a two-dimensional Kalman filter, consisting of a state (v_t) and its momentum (m_t). Observations are only directly dependent on the state v_t, but v_t is updated at each time step by addition of the momentum m_t (as indicated by the triangular state-transition matrix $\mathbf{F}$):

$$\mathbf{x}_t = \begin{bmatrix} m_t \\ v_t \end{bmatrix} \qquad \mathbf{F} = \begin{bmatrix} 1 & 0 \\ 1 & 1 \end{bmatrix} \qquad \mathbf{H} = \begin{bmatrix} 0 & 1 \end{bmatrix} \qquad \mathbf{Q} = \begin{bmatrix} q_m & 0 \\ 0 & q_v \end{bmatrix}$$

Substituting the above terms into equations (1) and (3) we get:

$$m_t = m_{t-1} + K_{t,1}\big((r_t - v_{t-1}) - m_{t-1}\big) \quad (8)$$

$$v_t = v_{t-1} + K_{t,2}\big(K_{t,3} m_{t-1} + r_t - v_{t-1}\big), \quad (9)$$

where $K_{t,1} = \frac{P_{t,1,1}+P_{t,1,2}}{q_v+\sum_{i,j} P_{i,j}}$ and $K_{t,2} = \frac{q_v+\sum_{i,j} P_{i,j}}{q_v+C+\sum_{i,j} P_{i,j}}$ are adaptive learning rates whose value is between 0 and 1, and $K_{t,3} = \frac{1}{K_{t,2}} - 1$ is an adaptive scaling factor.

Thus, the momentum estimate consists of a running average of recent outcome prediction errors, and the state estimate update includes a bonus that is proportional to the momentum estimate.

References

S1. Bishop, C.M. (2006) *Pattern Recognition and Machine Learning*, Springer
